# The effect of C_60_ fullerene on the mechanokinetics of *muscle gastrocnemius* contraction in chronically alcoholized rats

**DOI:** 10.1016/j.heliyon.2023.e18745

**Published:** 2023-07-27

**Authors:** Olexandr Motuziuk, Dmytro Nozdrenko, Svitlana Prylutska, Igor Vareniuk, Kateryna Bogutska, Serhii Braniuk, Olexandr Korotkyi, Yuriy Prylutskyy, Uwe Ritter, Jacek Piosik

**Affiliations:** a Faculty of Biology and Forestry, Lesya Ukrainka Volyn National University, Lutsk, 43025, Ukraine; b Department of Biophysics and Medical Informatics, ESC “Institute of Biology and Medicine”, Taras Shevchenko National University of Kyiv, Kyiv, 01601, Ukraine; c Department of Physiology, Plant Biochemistry and Bioenergetics, Faculty of Plant Protection, Biotechnology and Ecology, National University of Life and Environmental Science of Ukraine, Kyiv, 03041, Ukraine; d Institute of Chemistry and Biotechnology, Technical University of Ilmenau, Ilmenau, 98693, Germany; e Intercollegiate Faculty of Biotechnology, University of Gdansk, 80-307, Gdańsk, Poland

**Keywords:** С_60_ fullerene, *m. gastrocnemius*, Biomechanical and biochemical parameters, Histological analysis

## Abstract

The C_60_ fullerene effect (oral administration at a dose of 1 mg kg^−1^) on the selected biomechanical parameters of *muscle gastrocnemius* contraction, biochemical indicators of blood and muscle tissue as well as histological changes in rat muscle tissue after chronic alcoholization for 3, 6 and 9 months was studied in detail. Water-soluble C_60_ fullerenes were shown to reduce the pathological processes development in the muscle apparatus by an average of (35–40)%. In particular, they reduced the time occurrence of fatigue processes in muscle during the long-term development of alcoholic myopathy and inhibited oxidative processes in muscle, thereby preventing its degradation. These findings open up the possibility of using C_60_ fullerenes as potent antioxidants for the correction of the pathological conditions of the muscle system arising from alcohol intoxication.

## 1 Introduction

Chronic alcoholic myopathy develops in (40–60)% of alcohol abusers and is accompanied by decreased performance, proximal paresis, and skeletal muscle atrophy. It remains unclear whether the duration or amount of alcohol consumption is important for the development of chronic alcoholic myopathy. The chronic course of this pathological process in skeletal muscle is also unknown. Studies in men have shown that chronic alcoholic myopathy develops after 5 years of alcohol abuse [1].

Ethanol is a toxic substance, it causes direct and indirect effects on the muscular system, which leads to alcoholic myopathy. The metabolism of alcohol is closely related to enzymes involved in oxidative stress and the generation of reactive oxygen species (ROS), which cause damage to cells and tissues [2]. Oxidative stress can be considered the consequence of ROS formation imbalance and the antioxidant defense system. Alcohol is mainly metabolized in the liver by alcohol dehydrogenase enzymes. At the same time, ethanol oxidation, especially in patients with chronic alcoholism, also occurs with the participation of cytochrome P-450 2E1 [3]. Alcohol metabolism, ROS production, and disturbances in the redox state of cells are well-known pathways of tissue damage in several organ systems [4,5].

In rats that constantly consumed alcohol, a decrease in the activity of glutathione enzymes was observed [6]. In addition, skeletal muscles showed increased protein carbonylation [7], high levels of cholesterol hydroperoxide, and malondialdehyde (MDA) [8], indicating oxidative damage. In alcoholics, the excessive production of ROS by the microsomal system and mitochondria is noted [9]. Mitochondrial damage disrupts fatty acid oxidation and increases lipid peroxidation (LPO). Mitochondrial muscle damage was observed in 28% of alcoholics [10].

The activity of the antioxidant system plays a significant role in the pathogenesis of myopathy. Ethanol-treated rats showed an increase in superoxide dismutase (SOD) and glutathione peroxidase (GP_x_) activities, as well as MDA levels, which correlated with muscle iron content and type IIb fiber atrophy [11]. Zinc treatment did not reduce muscle atrophy in animals, although it did reduce MDA levels. Zinc, manganese, copper, and selenium are cofactors of antioxidant enzymes, while iron accumulation can contribute to LPO [12]. The authors [13] found a significant decrease in the levels of alpha-tocopherol and selenium in the blood of alcoholics with skeletal myopathy. At the same time, no significant changes in the levels of such antioxidants as alpha-tocopherol, ascorbic acid, and retinol were observed in blood and muscle tissue in patients with alcoholism with myopathy [14,15].

C_60_ fullerenes are known to be able to efficiently capture and inactivate free radicals in *in vitro* and *in vivo* systems [16,17]. C_60_ fullerene is more powerful antioxidant than natural antioxidant vitamin E in preventing the integrity of membranes from damage [18]. Recently we demonstrated, that water-soluble C_60_ fullerenes at low doses (0.1–1) mg kg^−1^ more effectively diminish the muscle fatigue in rats compared to the known exogenous antioxidants N-acetylcysteine and β-alanine [19,20], which are widely used in sport medicine. Moreover, they penetrate into cells and localize preferentially in mitochondria [21]. According to our previous data [22,23] water-soluble C_60_ fullerenes at concentrations up to 14.4 as well as 24 μg ml^−1^ did not manifest any toxic effects in rat erythrocytes and thymocytes as well as in human mesenchymal stem cells, respectively. It was shown that intraperitoneal administration of C_60_ fullerene suspension in a dose of 2.5 g kg^−1^ does not lead to mice death or to violations of their behaviour within 8 weeks [24]. It was established that the radiolabeled C_60_ fullerene and its derivative, fullerol, after intravenous administration to mice accumulate mainly in the liver, spleen, stomach, and blood and are excreted from the body within 72 h with mainly urine [25,26]. Our recent results [27] indicate the prolonged kinetics of water-soluble C_60_ fullerenes elimination from the body of rats, which contributes to their long-term (at least 48 h) compensatory activation of the endogenous antioxidant system in response to muscle stimulation.

In previous *in vivo* experiments, the administration of water-soluble C_60_ fullerenes has been shown to result in significant positive therapeutic effects following ischemic injury initiation [28,29], fatigue [30], atrophy [31], and injury [32] in skeletal muscles. It is clear that the observed effects depend significantly on the doses used and the pattern of the drug administration against the background of the initiation of a particular pathology.

Thus, the aim of the present study was to evaluate the effect of water-soluble C_60_ fullerenes as powerful exogenous antioxidants [16] on the magnitude of force response of *muscle gastrocnemius* of chronically alcoholized rats depending on the optimal dose and application pattern. In this context, it was also important to analyze biochemical indicators of blood and muscle tissue as markers of muscle damage [33], as well as histological changes of muscle tissue.

## 2 Materials and methods

### 2.1 Preparation of C_60_FAS

C_60_ fullerene aqueous solution (C_60_FAS) has been prepared according to Refs. [34,35]*.* The proposed method is based on transferring C_60_ molecules from the organic solution into the aqueous phase by ultrasonic treatment. The prepared C_60_FAS is a typical colloid fluid containing single C_60_ fullerenes (0.7 nm in diameter) as well as their nanoaggregates up to 100 nm [35,36]. The maximal concentration of C_60_ fullerenes in water obtained by this method was 0.15 mg ml^−1^.

The zeta potential is related to the stability of colloid dispersion. The zeta potential value for the C_60_FAS was −25 ± 2 mV [35,37]. A high negative surface charge of the individual nanoparticles (or, more strictly, the electrostatic repulsion between the negatively charged aggregates) indicates a very low tendency for them to aggregate over time in an aqueous solution (i.e., a high solute stabilization).

The C_60_FAS is stable within 18 months when stored at a temperature of +4 ^о^С.

### 2.2 *In vivo* experiment

The experiments were performed on male Wistar rats aged 1–10 months (at the end of the experiment). The study protocol was approved by the Bioethics Committee of the ESC “Institute of Biology and Medicine”, Taras Shevchenko National University of Kyiv in accordance with the rules of the “European Convention for the Protection of Vertebrate Animals Used for Experimental and Other Scientific Purposes” and the norms of biomedical ethics in accordance with the Law of Ukraine №3447 - IV February 21, 2006, Kyiv, “The Protection of Animals from Cruelty” during biomedical research.

The control group (n = 10) received 100% drinking water. Animals in the “alcoholization” experimental group (n = 10) were randomly selected. Each animal was placed in a separate cage to receive 40% ethanol in drinking water [38], which meant that the experimental groups had no access to 100% water until the full-dosed ethanol consumption [39]. Ethanol intake was calculated as 0.5% of the animal's body weight. The ethanol dose was recalculated every 24 h during the whole experiment [40]. Duration of alcoholization was 3, 6 and 9 months. The experimental animals of the “alcoholization + C_60_″ group (n = 10) together with alcohol orally received C_60_FAS at a dose of 1 mg kg^−1^ of rat weight. The C_60_FAS dose was recalculated every 24 h throughout the experiment. The amount of C_60_FAS administered was controlled by denying animals access to 100% drinking water until they had completely used the administered drug.

It is important to note that the selected dose of C_60_FAS (1 mg kg^−1^) in our experiments, as the most effective one, was chosen on the basis of previously conducted research [20,27,31]. Moreover, this dose does not present any toxicity: it is significantly lower than the LD_50_ value, which was 600 mg kg^−1^ body weight when administered orally to rats [41] and 721 mg kg^−1^ when administered intraperitoneally to mice [37].

Our previous studies [19] showed that there was no significant difference in the observed effects between different routes of C_60_FAS administration (oral and intraperitoneal). Therefore, we used in our research only the oral route of drug administration, as the most practical from the point of view of its further testing in the clinic.

The animals were anesthetized by intraperitoneal injection of nembutal (40 mg kg^−1^). Experiment preparation included cannulation (*a. carotis communis sinistra*) for drug administration and pressure measurement, tracheotomy, and laminectomy at the level of the lumbar spinal cord. The animals were euthanized using sodium thiopental overdose, and in some cases, cervical dislocation of the cervical vertebrae was used.

### 2.3 Biomechanical analysis of muscle contraction

A 12-bit analog-to-digital and digital-to-analog converter (ADC-DAC) was used to record the electrophysiological signals of muscle contraction. DAC output pulses were triggered by isolated stimulators (DS2A, Digitimer), which performed nerve stimulation. The input signals were fed through the amplifier (Brownlee) to the ADC and recorded with a sampling frequency of 10 kHz. A linear motor in the servo position was used to stretch the muscle and measure the force it developed. The efforts were measured using semiconductor strain gauges glued to rigid steel beams mounted on moving parts of the linear motor. The stiffness of the puller exceeded 0.06 N mm^−1^, and the time constant of transient processes did not exceed 60 ms [28].

The studied *muscle gastrocnemius* was freed from the surrounding tissues, the tendon part was cut in the distal part to the lower back. To prepare for the modulated stimulation of efferents in the corresponding segments, the ventral roots were cut directly at the places of their exit from the spinal cord. The filaments of the cut ventral roots were fixed on the stimulating electrodes and the stimulus sequence was cyclically distributed. Stimulation of efferents was carried out by electrical pulses of 2 ms duration, generated by a pulse generator. External load on the muscle was controlled using a system of mechanical stimulators [28].

When analyzing the myotic response of the studied muscle, several basic biomechanical parameters were analyzed as markers of the presence of certain link dysfunctions in the “excitation-response” chain [29,31,32,42], namely:

1. Change in the level of minimum muscle contraction force;
2. Change in the time to reach the maximum force response of the muscle;
3. Change in the level of the maximum force of muscle contraction;
4. Change in the integrated power of the muscle (calculated area under the force curve);
5. Change in the time of the reduction of muscle contraction force by 50% from the initial level as the indicator of muscle fatigue development at stimulation irritations;
6. Change in the time of muscle force response to the initial level.

### 2.4 Biochemical analysis

The levels of enzymes (creatine phosphokinase (CPK) and lactate dehydrogenase (LDH)) as well as creatinine and lactate (LA) in the blood plasma of the test animals and assessment of the level of oxidative processes in muscle tissues (content of hydrogen peroxide and reduced glutathione (GSH) as well as catalase (CAT), selenium-dependent GP_x_ and SOD activities), as markers of muscle damage [33], were determined using clinical diagnostic equipment (hemoanalyzers RNL-200 (Netherlands), ABX Micros ESV60 (France) and automatic analyzer Pentra C400 (France)).

Blood alcohol concentration (BAC) was determined at the end of the experiment by cardiac puncture using an AM1 alcohol analyzer (Analox Instruments Limited, UK).

### 2.5 Histological analysis

The samples of *muscle gastrocnemius* were separated and fixed in 10% formalin, embedded in paraffin, cut into 5 μm sections, and stained with hematoxylin and eosin (H&E) for general histopathological analyses, or with hematoxylin and picrofuchsin by van Gieson for detecting collagen fibers [43]. The histopathological profiles of each sample were determined by light microscopy observation. Also, digital microphotographs of stained sections were taken at × 400 magnification using a computer-assisted image analyzing system (it consists of an Olympus B×41 microscope and Olympus C-5050 Zoom digital camera, Japan). Then, the muscle fiber diameters and the area occupied by connective tissue in the muscle bundles were measured using ImageJ software.

### 2.6 Statistical analysis

Statistical processing of the measurement results was carried out by methods of variation statistics using the Origin 9.4 software. Each of the experimental kinetic curves is the result of averaging 10 similar measurements. No less than five repeats were performed for each biochemical and morphometrical measurement. Data are expressed as the means ± SEM for each group. The differences among experimental groups were detected by one-way ANOVA followed by Bonferroni's multiple comparison test. Values of *p* < 0.05 were considered significant.

## 3 Results and discussion

### 3.1 BAC value

**Fig. 1:**
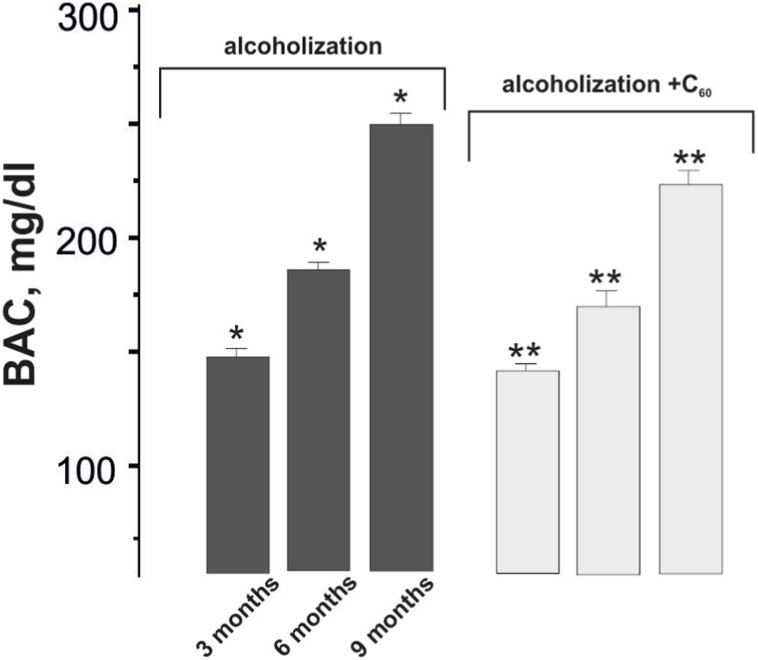
Blood alcohol concentration (BAC) in rats after chronic ethanol administration: alcoholization - rats treated with alcohol; alcoholization + C_60_ - rats treated with a mixture of alcohol and C_60_FAS at a dose of 1 mg kg^−1^ during the whole period of alcoholization; 3, 6 and 9 months - alcoholization lasting 3, 6 and 9 months, respectively. *p < 0.05; **p < 0.05 relative to the alcoholization group.

The BAC value in rats after chronic ethanol consumption ranged from 147 mg dl^−1^ (3 months of alcohol consumption) to 253 mg dl^−1^ (9 months of alcohol consumption) (Fig. 1). These data agree well with the results of a study [39].

C_60_FAS administration at a dose of 1 mg kg^−1^ in the therapeutic scheme (together with alcohol) did not result in a significant change in the BAC values throughout the experiment (Fig. 1).

### 3.2 Muscle contraction force analysis

**Fig. 2:**
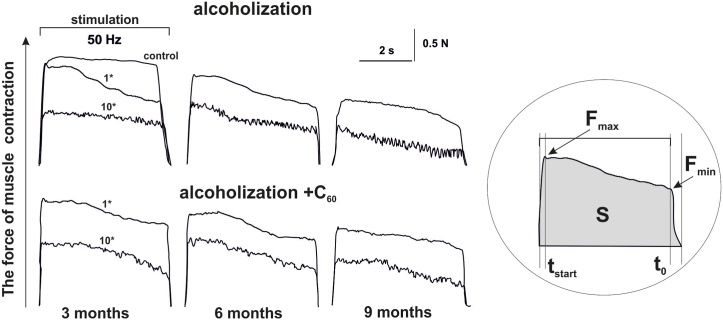
The forces of the 1st (1*) and 10th (10*) contractions of the *muscle gastrocnemius* of alcoholized rats induced by 6 s non-relaxation stimulation pools at 50 Hz: alcoholization - rats treated with alcohol; alcoholization + C_60_ - rats treated with a mixture of alcohol and C_60_FAS at a dose of 1 mg kg^−1^ during the whole period of alcoholization; 3, 6 and 9 months - alcoholization lasting 3, 6 and 9 months, respectively; F_max_ and F_min_ - the maximum and minimum levels of muscle force response; S - the integrated power of the muscle (calculated area under the force curve); t_start_ - the time of force reaching its maximum value; t_0_ - the time of force response reaching the initial level.

Fig. 2 shows the mechanograms of the 1st and 10th *muscle gastrocnemius* contraction in alcoholized rats at 50 Hz for 6 s stimulation. The recording of force responses reveals a sharp decrease in muscle force activity already at the 1st stimulus with a progressive decrease to (50–70)% of control values at the 10th one. A clear decrease in the amplitude of *muscle gastrocnemius* contraction in alcoholic rats was observed with increasing time of alcoholization from 3 to 6 and 9 months. In rats receiving alcohol and C_60_FAS (1 mg kg^−1^) together, an increase in the muscle force response was observed throughout the experiment compared to rats in the alcoholization group.

**Fig. 3:**
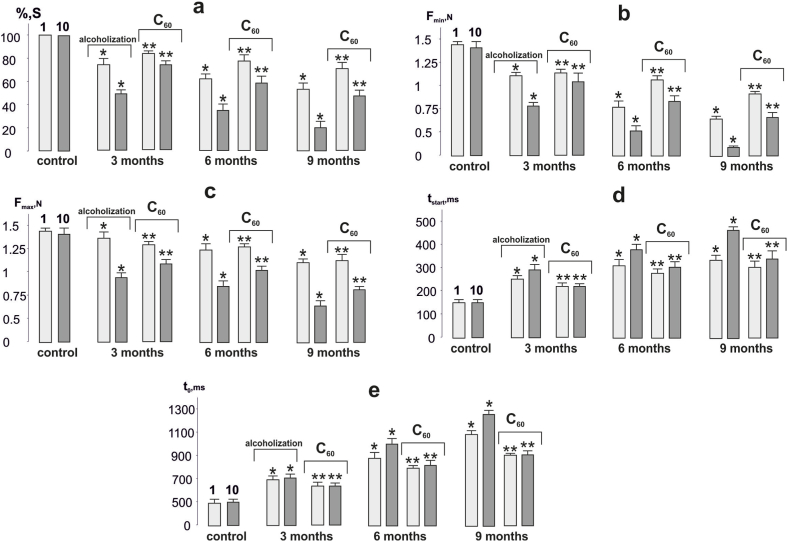
The levels of biomechanical markers of *muscle gastrocnemius* contraction in alcoholized rats when applied 6 s with non-relaxation stimulation at 50 Hz: alcoholization - rats treated with alcohol; C_60_ - rats treated with a mixture of alcohol and C_60_FAS at a dose of 1 mg kg^−1^ during the whole period of alcoholization; 1 and 10 - the strength of the 1^st^ and 10^th^ contractions, respectively; 3, 6 and 9 months - alcoholization lasting 3, 6 and 9 months, respectively; S - integrated muscle power (presented as a percentage of the maximum values) (a); F_min_ and F_max_ - minimum (b) and maximum (c) levels of muscle strength response; t_start_ - time of force reaching its maximum value (d); t_0_ - time of force response reaching initial level (e); *p < 0.05 relative to the control; **p < 0.05 relative to the alcoholization group.

For a more qualitative analysis of the findings, we analyzed changes in the main biomechanical markers of muscle activity (Fig. 3).

Integrated muscle power, as an overall muscle performance indicator, is one of the most important characteristics of muscle dysfunction. With 3, 6 and 9 months of alcoholization, it decreased at the 1^st^ contraction by 22 ± 2%, 29 ± 1%, and 34 ± 3%, respectively. It should be noted that at the 10^th^ contraction, this parameter decreased by 34 ± 3%, 56 ± 5%, and 74 ± 4%, respectively, compared to the 1st contraction (Fig. 3a). C_60_FAS administration resulted in an 11 ± 1%, 18 ± 1%, and 26 ± 2% increase in integrated muscle power at 3, 6 and 9 months of alcoholization, respectively. The difference between the 1st and 10th contractions was reduced to (15–39)%.

A change in minimum contraction force is one of the most sensitive markers of muscle dysfunction. Its decrease at the 1^st^ contraction was 35 ± 3%, 56 ± 3%, and 68 ± 5% with alcoholization of 3, 6 and 9 months, respectively (Fig. 3b). At the 10^th^ contraction, this parameter decreased by 56 ± 3%, 78 ± 4% and 91 ± 5% from the control values, indicating severe impairment of the studied muscle function. C_60_FAS administration increased this parameter by 9 ± 1%, 26 ± 2%, and 33 ± 3% with 3, 6 and 9 months of alcoholization, respectively.

A change in maximum contraction force levels is an indicator of overall muscle system dysfunction, indicating a reduction in the maximum possible force response during the development of pathology (Fig. 3c). At the 1^st^ contraction this index decreased slightly, namely by 8 ± 1%, 12 ± 1% and 21 ± 2%, with 3, 6 and 9 months alcoholization, respectively. However, at the 10^th^ contraction, this reduction was 47 ± 2%, 52 ± 3%, and 64 ± 5%, respectively, indicating a significant dysfunction of the maximal muscle force. The increase in maximal force contraction with C_60_FAS administration was 11 ± 1%, 17 ± 1%, and 23 ± 2% with 3, 6 and 9 months of alcoholization, respectively.

The increase in the time to maximum force response indicates the level of physiological dysfunction of the muscle apparatus when it implements its maximum force tasks during precise positional movements (Fig. 3d). Its increase at 3, 6 and 9 months of alcoholization was 31 ± 3%, 44 ± 4% and 49 ± 4%, respectively, at the 1^st^ contraction with a further increase of (25–30)% at the 10^th^ one. The correction for this parameter with C_60_FAS administration was (15–20)%. Note that in this case the difference between the 1st and 10th contractions was reduced to (5–8)%.

The change in muscle force response release time to the initial level is associated with an increase in intramuscular collagen structures, an increase in subfascial pressure and the presence of non-functioning muscle fibers. The studied parameter describes a change in muscle stiffness associated with both an increase in connective tissue components of the muscle (long-term pathology) and a change in intramuscular pressure (acute period of pathology). Its increase was 48 ± 4%, 88 ± 8%, and 123 ± 8% with 3, 6 and 9 months of alcoholization, respectively, at the 1^st^ contraction, with a (9–12)% increase at the 10^th^ one. C_60_FAS administration reduced this parameter by 15 ± 1%, 29 ± 2%, and 41 ± 3%, respectively. The difference between the 1^st^ and 10^th^ contractions was reduced to (3–5)%.

Thus, the proposed therapeutic regimen of C_60_FAS administration (1 mg kg^−1^; together with alcohol) leads to significant positive biomechanical changes in the *muscle gastrocnemius* contractile processes, namely, the level of muscle damage severity is reduced by an average of (35–40)%.

**Fig. 4:**
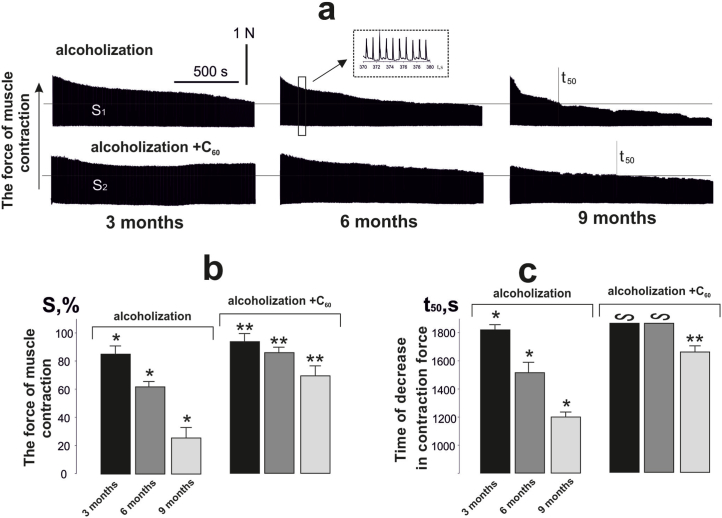
*Muscle gastrocnemius* contraction force of alcoholized rats induced by 1 Hz relaxation-free stimulation lasting 1800 s (a), and biomechanical parameters of fatigue development: S - integrated muscle power (b); time to 50% decrease in force response from initial values (t_50_) (c); alcoholization - rats treated with alcohol; alcoholization + C_60_ - rats treated with a mixture of alcohol and C_60_FAS at a dose of 1 mg kg^−1^ during the whole period of alcoholization; 3, 6 and 9 months - alcoholization lasting 3, 6 and 9 months, respectively; *p < 0.05; **p < 0.05 relative to the alcoholization group.

Recording the contractile force of rat *muscle gastrocnemius* after 3, 6 and 9 months of alcoholic induction with 1 Hz fatigue stimulation for 1800 s (Fig. 4a) showed a decrease in integrated muscle power of 82 ± 5%, 60 ± 3%, and 22 ± 1% of control values (Fig. 4b). Administration of C_60_FAS to experimental animals changed this to 94 ± 4%, 85 ± 2%, and 69 ± 5% (Fig. 4b). The 50% reduction in force response time from initial values (t_50_) was 1740 ± 25 ms, 1460 ± 91 ms, and 1220 ± 25 ms (Fig. 4c). When C_60_FAS was administered to rats subjected to 3 and 6 months of alcoholization, no 50% reduction in force response was observed (Fig. 4c). With alcoholization lasting 9 months, this parameter was 1620 ± 28 ms. Thus, it can be argued that the proposed therapeutic regimen of C_60_FAS administration (dose 1 mg kg^−1^; together with alcohol) reduces the time of occurrence of fatigue processes in alcohol-induced muscle and is most effective specifically in the long-term development of alcoholic myopathy.

The results obtained indicate that the consequence of alcoholic myopathy is the deterioration of contractile muscle activity. The reason for this is both ultrastructural changes of myocytes and their atrophy, and changes in electrolyte homeostasis and bioelectric activity in general [44]. During the development of alcoholic myopathy, first of all, there is a hypergeneration of free radicals in dysfunctional mitochondria [45,46], which causes the destruction of the membrane structures of myocytes and functional disorders of their enzyme systems [47]: a decrease in the activity of Na^+^/K^+^-ATPase and an increase in ‒ Ca^2+^-ATPase. The miotic disorders cause an imbalance in the correction of the joints positioning and progressive muscle weakness, which leads to the degradation of muscle strength with 3, 6 and 9 months of alcoholization. From a functional point of view, this indicates that a large amount of high-energy phosphate compounds is consumed by the damaged muscle cell to maintain homeostasis and, as a consequence, there is a metabolic disturbance, leading to a significant increase in muscle fatigue with the development of alcoholic myopathy. At the same time, the use of C_60_FAS significantly increases the energy capacity of actively contracting alcohol-induced muscle.

### 3.3 The analysis of biochemical parameters in blood plasma and muscle tissue

**Fig. 5:**
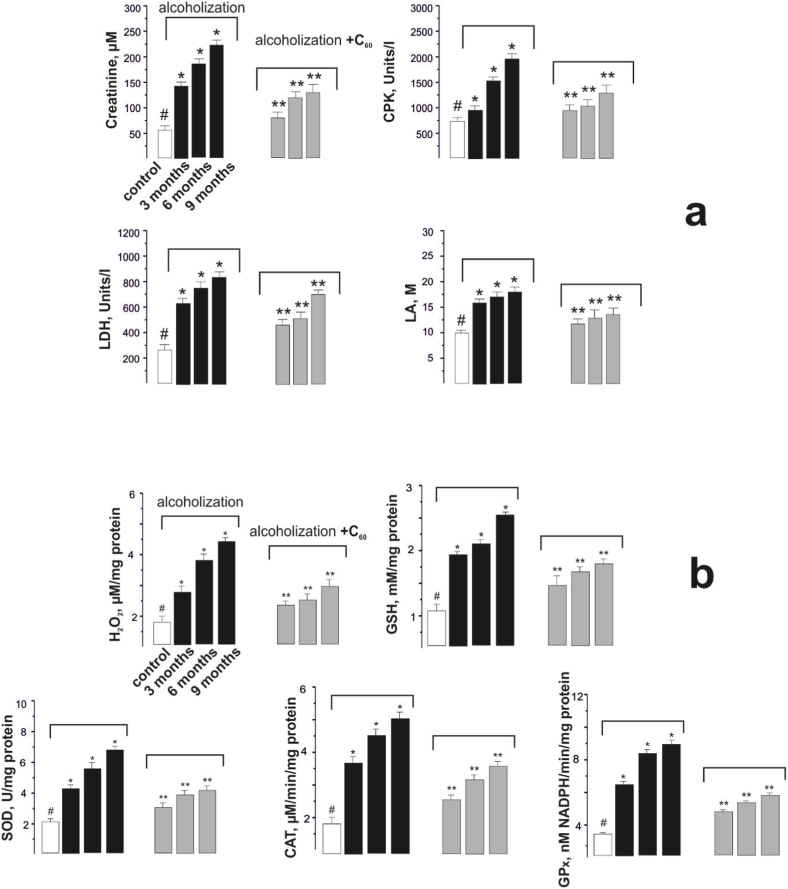
The content of creatinine, CPK, LDH, and LA in the blood plasma of alcoholized rats (a) and indicators of pro- and antioxidant balance (H_2_O_2_, GSH, SOD, CAT, and GP_x_) in rat *muscle gastrocnemius* (b): alcoholization - rats treated with alcohol; alcoholization + C_60_ - rats treated with a mixture of alcohol and C_60_FAS at a dose of 1 mg kg^−1^ during the whole period of alcoholization; 3, 6 and 9 months - alcoholization lasting 3, 6 and 9 months, respectively; *p < 0.05 relative to the control; **p < 0.05 relative to the alcoholization group.

To confirm the biomechanical results obtained, we analyzed blood plasma biochemical indices, which are used to diagnose the development of muscle myopathies [33]. In particular, changes in creatinine, CPK, LA, and LDH levels made it possible to assess the physiological state of *muscle gastrocnemius* against the background of prolonged alcoholization of animals (Fig. 5).

It should be noted that the above biochemical markers have a pronounced tendency to increase with the increasing duration of alcoholization of animals. In cases where rats received a mixture of alcohol and C_60_FAS, these indices were significantly lower compared to alcohol-impaired animals. Thus, creatinine was reduced by 16 ± 1%, 18 ± 1%, and 37 ± 2%, CPK by 13 ± 1%, 18 ± 1%, and 28 ± 2%, LDH by 15 ± 1%, 29 ± 2% and 41 ± 2%, and LA by 19 ± 1%, 27 ± 2% and 38 ± 2% when alcohol and C_60_FAS were co-administered and alcoholization lasted 3, 6 and 9 months, respectively (Fig. 5a). Thus, the observed changes in biochemical indicators in the blood plasma of tested animals correlate well with the biomechanical parameters of *muscle gastrocnemius* functioning described above.

The obtained positive therapeutic effect of water-soluble C_60_ fullerenes can be explained by their powerful antioxidant properties [19,41]. The determination of changes in the pro- and antioxidant balance in the tissues of the muscle under study is essential to confirm this hypothesis [48].

H_2_O_2_ concentration increased with the increasing duration of alcoholization and was 151 ± 7%, 327 ± 9%, and 414 ± 11% when alcoholization lasted 3, 6 and 9 months, respectively, compared with the control muscle. Co-administration of alcohol and C_60_FAS resulted in 28 ± 2%, 44 ± 2%, and 51 ± 2% reductions in H_2_O_2_ and GSH by 13 ± 1%, 27 ± 1%, and 42 ± 2%, activities of SOD by 21 ± 1%, 29 ± 1% and 44 ± 2%, CAT by 12 ± 1%, 34 ± 2% and 48 ± 2%, and GP_x_ by 17 ± 1%, 23 ± 1% and 39 ± 2% during alcoholization of 3, 6 and 9 months, respectively (Fig. 5b). It is important to note that the increase in muscle GSH content due to C_60_FAS increases resistance to exercise [30]. Thus, in all tests there is a positive change in the pro- and antioxidant biochemical balance in rat *muscle gastrocnemius* by about (15–30)% when C_60_FAS is administered at a dose of 1 mg kg^−1^, together with alcohol.

Thus, based on the data obtained, it can be concluded that the application of water-soluble C_60_ fullerenes contributes to the reduction of oxidative processes in muscle by maintaining the balance between pro-oxidants and antioxidant defense system, prevents the negative effect of ROS on cellular and subcellular structures in the development of alcoholic myopathy in rats. In our opinion, C_60_ fullerene affects the activity of endogenous antioxidants, suppressing the occurrence of destruction in muscle and thus reducing its degradation.

### 3.4 Histological analysis of muscle tissue

**Fig. 6:**
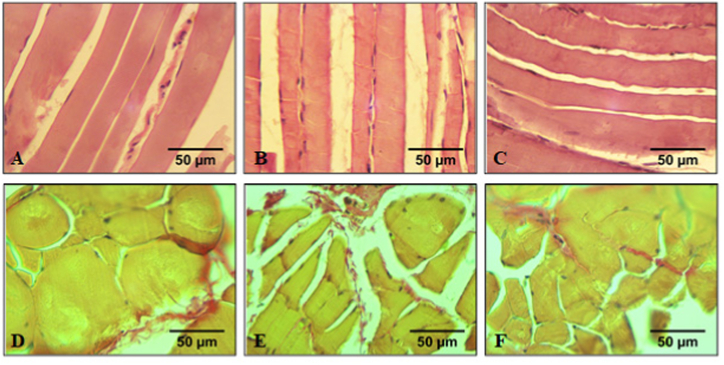
Representative histological images of *muscle gastrocnemius*: a and d - control group; b and e − alcoholization group; c and f - alcoholization + C_60_ group. a,b and c – hematoxylin and eosin staining; d,e and f - hematoxylin and picrofuchsin staining by van Gieson. Scale bar - 50 μm.

In alcoholized rats, most of the muscle fibers are thinner than in the control group (Fig. 6a and d), so the average diameter of the fibers is reduced to 24.4 μm (Table 1). There are tortuous fibers. Some of the fibers have an uneven thickness along the fiber. Some fibers are vacuolized, which indicates destructive processes in them. In some fibers, the transverse striation is less clear, which may indicate a violation of the correct spatial arrangement of actin-myosin complexes. Within thin fibers, there are also fibers of normal thickness (Fig. 6b). Connective tissue between muscle fibers is enlarged (Fig. 6e). As a result, the area occupied by connective tissue increase up to 0.21 μm^2^ per 1 μm^2^ cross-sectional area of the muscle (Table 1). So, sclerosis and fibrosis are observed.

**Table 1:** Morphometrical data of *muscle gastrocnemius* in rats after alcohol and C_60_FAS consumption.

Group	Diameter of muscle fibers, μm	Area occupied by connective tissue, μm^2^/μm^2^
Control	36.7 ± 1.3	0.15 ± 0.02
Alcoholization	24.4 ± 1.4*	0.21 ± 0.02*
Alcoholization + C_60_	32.8 ± 2.5**	0.17 ± 0.02**

*p < 0.05 compared with control; **p < 0.05 compared with alcoholization group.

In alcoholized rats that received C_60_FAS, the thickness of the muscle fibers is not reduced compared to the control group. Sclerosis and fibrosis are lesser. So, the pathohistological manifestations in this group of rats are smaller (Table 1; Fig. 6c and f).

Summarizing, the following mechanism of C_60_ fullerene action can be considered. It is known that more than 90% of consumed alcohol is metabolized by oxidative and non-oxidative pathways, producing such chemically active compounds as acetaldehyde, acetate, fatty acid ethyl ester, etc. [49]. These compounds generate ROS, which cause increased oxidative stress and LPO, disrupting thus the structural integrity of cells and tissue functions in general. Therefore, C_60_ fullerenes, as powerful antioxidants [16,17], effectively absorbing ROS, normalize the functional state of the muscular system against the background of alcoholic myopathy.

## 4 Conclusions

Thus, the work demonstrated for the first time in animal models that the level of chronic alcoholic myopathy development decreased when water-soluble C_60_ fullerene at a dose of 1 mg kg^−1^ was used as a therapeutic nanoagent, which was confirmed by the biomechanical markers analysis of the contractile process of *muscle gastrocnemius*. The proposed combined administration of alcohol and C_60_FAS leads to a decrease in the time of fatigue processes in alcohol-induced muscle and is most effective precisely in the long-term development of alcoholic myopathy. The analysis of blood plasma biochemical parameters, changes in pro- and antioxidant balance, and histological indices in the tissues of the studied *muscle gastrocnemius* make it possible to state that the administration of water-soluble C_60_ fullerenes as potent antioxidants prevents the negative effects of ROS on cellular and subcellular structures in alcoholic myopathy development. This opens up new possibilities in the therapy of the pathological conditions of the muscular system, which are based on the pathological action of free-radical processes.

## Author contribution statement

Olexandr Motuziuk, Dmytro Nozdrenko, Svitlana Prylutska, Igor Vareniuk, Kateryna Bogutska, Serhii Braniuk, Olexandr Korotkyi, Yuriy Prylutskyy, Uwe Ritter and Jacek Piosik: Conceived and designed the experiments; Performed the experiments; Analyzed and interpreted the data; Contributed reagents, materials, analysis tools or data; Wrote the paper.

## Data availability statement

Data will be made available on request.

## Additional information

No additional information is available for this paper.

## Declaration of competing interest

The authors declare that they have no known competing financial interests or personal relationships that could have appeared to influence the work reported in this paper.
